# Unveiling aphasia: Bilateral anterior temporal lobe atrophy mimicking psychosis

**DOI:** 10.5339/qmj.2025.116

**Published:** 2025-12-14

**Authors:** Javed Latoo, Ovais Wadoo, Imtiaz Hussain Mansur, Yousaf Iqbal, Nabil Sherif Mahmood, Mohammed Ibrahim Alhatou, Majid Alabdulla

**Affiliations:** 1 Department of Psychiatry, Hamad Medical Corporation, Doha, Qatar; 2 College of Medicine, Qatar University, Doha, Qatar; 3 Department of Radiology, Hamad Medical Corporation, Doha, Qatar; 4 Department of Neurology, Hamad Medical Corporation, Doha, Qatar *Email: OWadoo@hamad.qa

**Keywords:** Aphasia, Wernicke aphasia, psychotic disorders, schizophrenia

## Abstract

**Introduction:**

Language impairment can present as a symptom of both psychiatric or a neurological condition, so making an accurate diagnosis is essential for appropriate management. While primary psychiatric disorders such as schizophrenia may include thought disorganization and speech abnormalities, true language dysfunction is typically associated with neurological pathology, such as stroke, neurodegenerative diseases, or traumatic brain injury. In cases where patients present with language impairment but lack a clear medical and psychiatric history, distinguishing between these possibilities becomes particularly challenging. A comprehensive assessment, including a neurological examination with potential imaging and a psychiatric evaluation, is crucial in these scenarios. This challenge is even more pronounced in populations such as lower-skilled migrant workers, where language barriers and a lack of collateral information further complicate the diagnostic process.

**Case Presentation:**

We report on a case of a patient presenting with significant language impairment who was initially misdiagnosed with a psychotic illness. The patient exhibited speech disturbances and communication difficulties that were initially interpreted as signs of disorganized thought processes, a hallmark of psychosis. However, further evaluation, including neuroimaging, revealed significant atrophy in the bilateral anterior temporal lobes, confirming a neurological basis for the symptoms. The absence of a prior medical history and limited collateral information contributed to the initial misdiagnosis.

**Conclusion:**

This case highlights the critical need for a thorough psychiatric and neurological workup in patients presenting with language impairment and an unclear history. Misdiagnosis can lead to inappropriate treatment and worsen patient outcomes. A careful evaluation, including neuroimaging and linguistic assessment, is essential in distinguishing between psychiatric and neurological etiologies. Moreover, addressing barriers related to language and medical history is vital to improving diagnostic accuracy, particularly in immigrant populations.

## 1. INTRODUCTION

Aphasia predominantly manifests in individuals following cerebrovascular accidents but may also arise in neurodegenerative conditions, such as Alzheimer’s disease, frontotemporal lobar degeneration, vascular dementia, brain tumors, or traumatic brain injuries.^1^ Approximately one-third of aphasia cases are linked to cerebrovascular accidents.^2^ Stroke patients typically exhibit aphasia and varying degrees of neurological impairment, particularly when damage occurs in the left cortical hemisphere.^3^ Diagnostic evaluation of aphasia commonly involves initial assessment with non-contrast computed tomography (CT) to ascertain potential cerebrovascular accident, followed by magnetic resonance imaging (MRI) for precise lesion localization. In cases where a new patient presents with incomprehensible speech-like aphasia without evident neurological abnormalities upon examination and normal CT findings, psychotic disorders may be considered, prompting psychiatric evaluations. This attribution stems from the resemblance between the speech patterns observed in aphasic patients and those in individuals with schizophrenia or other psychotic disorders.

The diagnostic challenge is particularly significant in Qatar, where foreign nationals constitute over 80% of the 2.7 million population, with lower-skilled migrant workers (mainly single male laborers) making up nearly half.^4^ Qatar’s healthcare system is largely state-funded, with primary care for low-income migrant workers provided by the Qatar Red Crescent Society and secondary care, including mental health services, managed by Hamad Medical Corporation.^5,6^ Although psychiatric care, including access to medications and both inpatient and outpatient services, is either free or highly subsidized, migrant workers often face difficulties in accessing healthcare. Factors such as language barriers, cultural differences, transportation issues, financial concerns, and mental health stigma contribute to delayed or missed diagnoses. These challenges are particularly relevant in cases where language impairments are mistakenly attributed to psychiatric disorders, increasing the risk of misdiagnosis and inappropriate treatment.^7–9^ Numerous initiatives and legislative changes have been implemented over time to enhance access to care.^10–19^

This case report was approved for publication by the institutional review board under approval number: MRC 04-24-840. The approval process ensured that all ethical considerations were met, including patient confidentiality and informed consent, in line with the ethical standards required for the publication of clinical case reports.

## 2. CASE PRESENTATION

The patient, a middle-aged male, was brought to the Emergency Department (ED) of our hospital by the police and Emergency Medical Services (EMS). It is not clear why the police initially stopped the patient, but it appears that the stop was part of a routine identity check rather than being related to any violent or aggressive behavior. Upon interaction, the patient was unable to engage in coherent conversation, and his speech was incomprehensible, which raised concerns for his welfare. The police officers, unsure of the cause of his communication difficulties and worried about his well-being, requested EMS to transport the patient to the hospital for further evaluation.

In the ED, it was established that the unknown male is a 45-year-old, Indian male who is employed as a low-skilled worker. His psychiatric history, full medical history, and baseline function were unknown, as attempts to contact reliable informants proved unsuccessful. He was unable to respond coherently. His speech was irrelevant, and despite efforts to communicate with the patient in his native language, there were significant challenges in communication. On presentation, vital signs were within normal limits. He had a heart rate of 84 bpm, a respiratory rate of 18/min, and a blood pressure of 160/112 mm Hg. His temperature was 36.5°C, and SpO^2^ was 100 %. There were no symptoms reported or systemic signs elicited during the assessment. Full blood count and biochemical profile results were within normal limits, as was the urinary drug screening. However, serum creatinine levels were moderately elevated at 139 mmol/L. The SARS-CoV-2 test was negative. A non-contrast CT scan of the brain was performed, and the results were normal, showing no evidence of any acute neurological abnormalities. Given the elevated creatinine level and hypertension, the initial working diagnosis of Delirium was made by the medical team. He was started on antihypertensive medication. The diagnosis of delirium is clinical, and delirious patients often are confused and unable to provide accurate information. The diagnostic investigations included cerebrospinal fluid (CSF) polymerase chain reaction and kidneys, ureters, and bladder (KUB) ultrasound, both of which were normal. Neurology and psychiatry consultations were also requested for further evaluation.

On a subsequent medical review, the patient’s speech continued to be incomprehensible; it was difficult to ascertain whether he met the diagnostic criteria for delirium. Given his creatinine levels were within normal limits and there were no systemic symptoms or signs, abnormal laboratory results, or neurological findings that could account for delirium, the patient was transferred to psychiatric care for further evaluation.

During his admission to a psychiatric ward, a friend of his was able to provide some information on his medical history and baseline function. The collateral information revealed that the patient originates from a village in India, where he lived in a close-knit community. There was no history of diagnosed medical or psychiatric illness. He moved to Qatar some years ago with acquaintances from his village. He was involved in a manual job without having to interact with others. He was fully dependent on these men from his village. He was unable to use public transportation independently. He had a limited understanding of finances and relied on these men to send money to his family. Even traveling back to India required accompaniment by a friend. Over the last year or so, he started having language difficulties.

On the psychiatric ward, the patient exhibited rapid, irrelevant speech resembling word salad. He ate and slept normally. A provisional diagnosis of “psychotic disorder not otherwise specified” was made. He was started on antipsychotics. There was no change in his presentation whilst being maintained on antipsychotics. The psychiatrists requested an MRI and an electroencephalogram (EEG).

**Figure 1:**
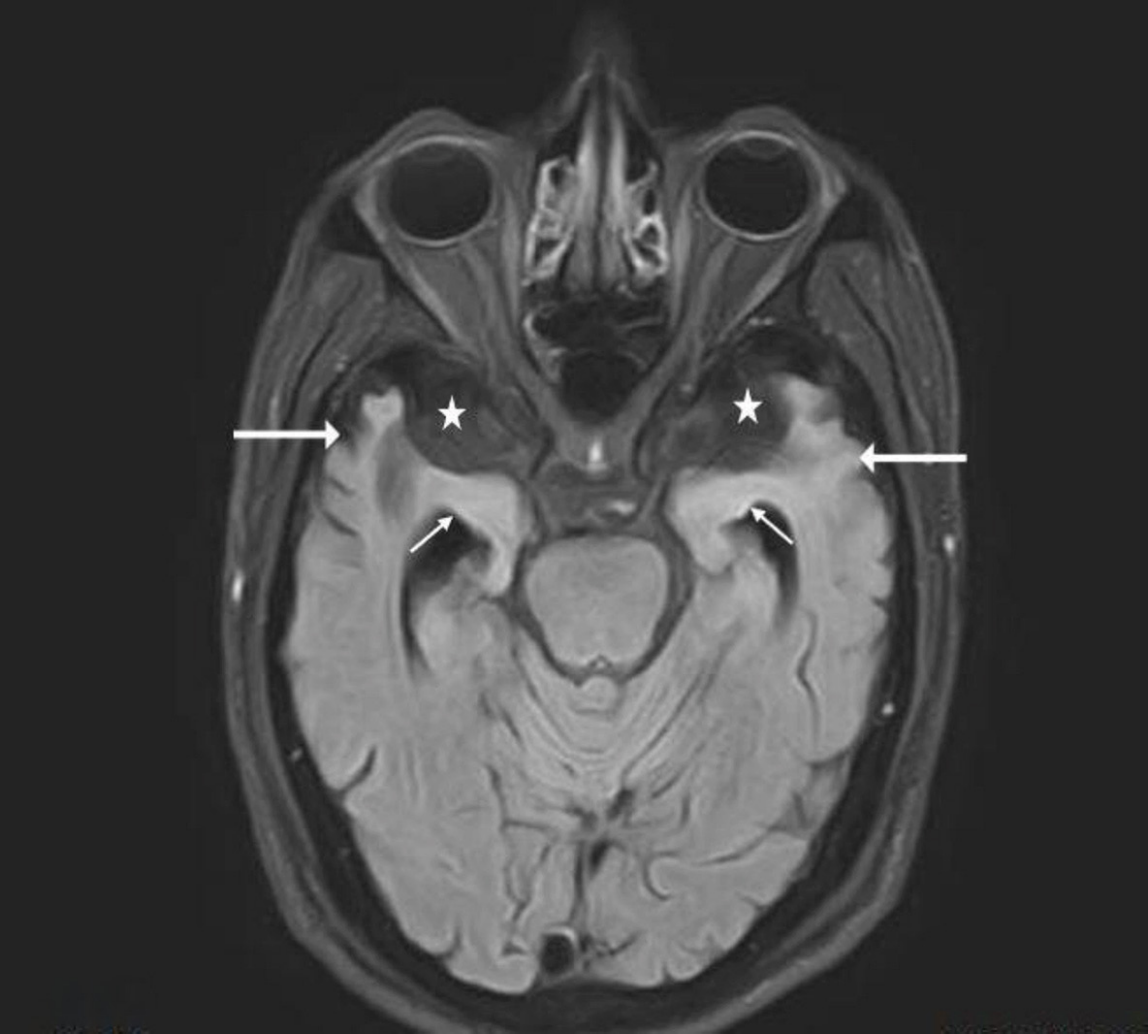
Axial FLAIR sequence of the brain at the level of the midbrain showing dilated extra-axial CSF spaces in the middle cranial fossa anteriorly (white asterisks), along with ex vacuo dilatation of the temporal horns of both lateral ventricles (short white arrow) with atrophy of both temporal lobes (long white arrows). FLAIR: Fluid atenuated inversion recovery.

**Figure 2:**
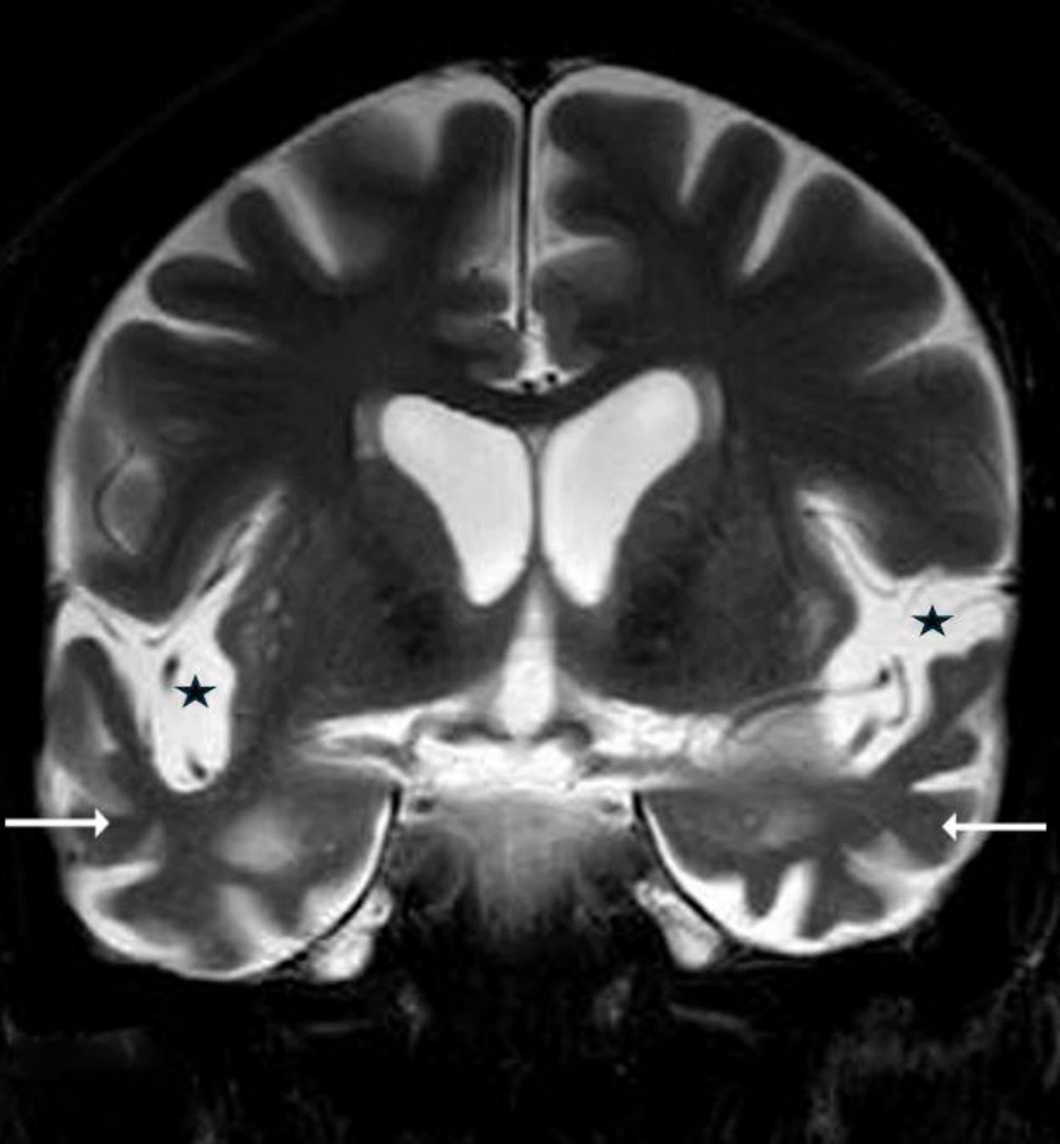
Coronal T2W sequence of the brain showing widened sylvian fissures on both sides (black asterisks) with atrophy of both temporal lobes (long white arrows). T2W: T2-weighted.

MRI of the head revealed significant atrophy in the bilateral anterior temporal lobes with prominent sylvian fissures and adjacent extra-axial CSF space, along with dilatation of the temporal horns, suggesting a possible neurodegenerative disease. The patient was diagnosed provisionally with Wernicke’s Aphasia (fluent aphasia) secondary to atrophy in the bilateral anterior temporal lobes. EEG was normal. The antipsychotic was stopped, and an evaluation by the speech and language therapist was requested. The patient requested a discharge even before further assessments were carried out. The patient left the country after discharge, and no further information on his progress or follow-up was available. This is a common practice in Gulf countries, where many migrant workers often return to their home countries after receiving initial medical treatment. While this is understandable, it limits the ability to gather long-term follow-up data (Figures 1 and 2).

## 3. DISCUSSION

Fluent aphasics and individuals with schizophrenia are noted for their speech patterns characterized by the use of meaningless and disjointed words. In psychosis, speech tends to be disorganized, often straying off-topic, lacking coherence, and proving challenging to follow. It may exhibit vagueness, repetition, and a reduction in syntactic and lexical complexity. Moreover, vocal characteristics often include a flat affect, with emotionally intense thoughts expressed in a disconnected manner.^20^ Conversely, aphasia affects language expression, comprehension, reading, and writing abilities. Fluent aphasic individuals may produce lengthy, grammatically correct sentences devoid of meaningful content, incorporating unnecessary or invented words, thereby hindering communication comprehension.^21^ Consequently, distinguishing between the speech of fluent aphasics and some schizophrenia patients can be challenging due to their shared characteristics.

In our patient with no previous medical or psychiatric history available and no apparent neurological insult, the diagnosis of psychosis was considered due to incomprehensible speech. However, further assessment, including the absence of positive and negative symptoms characteristic of schizophrenia, along with radiological evidence revealing atrophy in the bilateral anterior temporal lobes, prompted consideration of a language disorder. It becomes imperative to conduct a comprehensive language evaluation, encompassing tasks such as following commands, responding to inquiries, object naming, and engaging in conversation to gauge language production and comprehension abilities. When aphasia is suspected, referral to a speech-language therapist is warranted. The speech-language therapist conducts a thorough examination, assessing the individual’s capacity for verbal expression, social interaction, language comprehension, and literacy skills.

Aphasia arises from brain damage affecting language-processing regions, typically located in the left hemisphere.^1,7^ It often manifests suddenly, commonly following a stroke or head trauma, although a gradual onset may occur due to factors such as brain tumors or progressive neurological conditions. This disorder compromises language expression, comprehension, as well as reading and writing abilities. Aphasia is broadly classified into two categories: fluent and nonfluent, each comprising several subtypes.^22,23^ Damage to the temporal lobe frequently leads to Wernicke’s aphasia, the predominant form of fluent aphasia.^24^ Individuals with Wernicke’s aphasia encounter challenges in understanding speech and may produce lengthy, grammatically correct sentences devoid of meaning, incorporating unnecessary or invented words. Consequently, communication comprehension becomes arduous. Importantly, individuals with Wernicke’s aphasia often lack awareness of their verbal errors.

Conversely, damage to the frontal lobe of the brain can lead to Broca’s aphasia, the primary form of nonfluent aphasia. Individuals with Broca’s aphasia may experience right-sided weakness or paralysis of the arm and leg, as the frontal lobe also governs motor functions. While they may comprehend speech and possess the intention to communicate, they often struggle to articulate themselves, typically producing short phrases with considerable effort. Commonly, they omit small words, such as “is,” “and,” and “the.” Despite these challenges, individuals with Broca’s aphasia generally maintain a relatively intact understanding of others’ speech. Consequently, they often recognize their communication difficulties and may become easily frustrated.^25^

Global aphasia, another variant, stems from extensive damage to multiple language-processing regions of the brain.^26^ Individuals with global aphasia face profound communication challenges, often exhibiting severe limitations in speech production and comprehension. They may struggle to utter even a few words or may repetitively echo specific words or phrases. Understanding simple language, too, proves challenging for them. Importantly, aphasia excludes (a) developmental language disorders (DLDs), (b) solely motor speech disorders, which affect speech articulation via the oral-motor apparatus, such as stuttering, dysarthria, and apraxia of speech, or (c) language disorders arising secondarily from primary thought disorders like schizophrenia.

DLD was excluded as a diagnosis. DLD represents a communication disorder disrupting the acquisition, comprehension, and utilization of language.^27,28^ These language impairments are not attributable to other conditions such as hearing loss or autism, nor to situational factors like limited exposure to language. DLD can impede a child’s speech, listening, reading, and writing abilities. Previously known as specific language impairment, language delay, or developmental dysphasia, DLD is among the most prevalent developmental disorders, affecting approximately 1 in 14 children. Its effects can persist into adulthood. MRI findings associated with DLD are variable, ranging from normal results to indications of ventricular enlargement, central volume loss, and white matter abnormalities.^29^ Such findings suggest that DLD may signify broader dysfunction within the nervous system.

Another potential consideration for differential diagnosis was Semantic Dementia (SD), also known as the temporal variant of Frontotemporal Lobar Degeneration. SD is characterized by fluent, anomic aphasia and behavioral alterations accompanied by pronounced, often asymmetrical degeneration of the anterior temporal lobes.^30–32^ In cases with predominant left-sided atrophy, initial symptoms typically entail progressive deterioration of semantic knowledge related to words, objects, and concepts. This presents as fluent aphasia with impoverished speech content and semantic paraphasic errors, yet preserved syntax, prosody, and motor speech. Semantic loss follows a hierarchical pattern, initially affecting specific distinctions (e.g., types of dogs) before progressing to general categorization difficulties (e.g., distinguishing animals from other objects). Eventually, semantic impairment extends beyond language, leading to features of multimodal agnosia. Neuropsychological assessments commonly reveal deficits in confrontation naming, word-to-picture matching, and category fluency, while episodic memory (particularly visual memory), spatial abilities, and executive functions remain relatively intact. Notably, left-sided SD cases predominate over right-sided cases in most instances, although this may be influenced by referral biases favoring the identification of left-sided cases in behavioral neurology centers, potentially leading to misdiagnosis of right-sided cases as primary psychiatric disorders. SD in our patient was ruled out as it classically affects adults in their fifties to sixties.^33^ The nature of language impairment did not support the diagnosis. Bilateral neurodegeneration of the anterior temporal lobes is associated with profound lexical-semantic deficits, yet syntax is strikingly spared.^34,35^

Another potential differential to consider was Primary Progressive Aphasia (PPA).^36–38^ However, PPA was excluded from consideration as it typically manifests with isolated aphasia without other cognitive impairments and lacks the characteristic radiological features of marked atrophy in the left temporal lobe.

## 4. CONCLUSION

This case highlights the diagnostic complexity of distinguishing between psychiatric disorders and language impairments, particularly in linguistically and culturally diverse populations. In migrant worker populations with limited access to healthcare and no reliable history, careful clinical assessment supported by neuroimaging is essential. Misattributing aphasia to psychosis can delay appropriate intervention. Clinicians should maintain a high index of suspicion for neurodegenerative causes of language disturbance and ensure timely involvement of neurology and speech-language pathology services.

## CONFLICT OF INTERESTS

The authors declared no potential conflicts of interest with respect to the research, authorship, and/or publication of this article.
